# Association of e-Cigarette Advertising, Parental Influence, and Peer Influence With US Adolescent e-Cigarette Use

**DOI:** 10.1001/jamanetworkopen.2022.33938

**Published:** 2022-09-29

**Authors:** Yu Wang, Zongshuan Duan, Scott R. Weaver, Shannon R. Self-Brown, David L. Ashley, Sherry L. Emery, Jidong Huang

**Affiliations:** 1 School of Public Health, Georgia State University, Atlanta; 2 Milken Institute School of Public Health, George Washington University, Washington, District of Columbia; 3 NORC at University of Chicago, Chicago, Illinois

## Abstract

**Importance:**

Little is known about the roles of advertising and parental and peer influence in e-cigarette use among US adolescents in recent years, hindering efforts to address the increasing rate of youth vaping.

**Objective:**

To examine how e-cigarette advertising exposure and parental and peer e-cigarette use were associated with e-cigarette use among US adolescents.

**Design, Setting, and Participants:**

This cohort study used data from waves 4 (December 2016 to January 2018), 4.5 (December 2017 to December 2018), and 5 (December 2018 to November 2019) of the Population Assessment of Tobacco and Health study, an on-going cohort study representative of the noninstitutionalized US population. Sample weights were applied to generate nationally representative estimates. Data were analyzed in January 2022.

**Exposures:**

Past 30-day e-cigarette advertising exposure, past 30-day parental e-cigarette use, and the number of best friends using e-cigarettes (none, a few, some, most, and all).

**Main Outcomes and Measures:**

Outcomes were contemporary curiosity about using e-cigarettes and e-cigarette initiation at follow-up. Generalized estimating equations were used to estimate the weighted adjusted associations.

**Results:**

Wave 4 included 8548 adolescents; wave 4.5, 10 073 adolescents; and wave 5, 11 641 adolescents. Among adolescents in the wave 4 survey, 4425 (51.1%) were boys, 1935 (24.9%) were aged 12 years, 1105 (13.0%) were Black, 2515 (24.4%) were Hispanic, and 3702 (52.3%) were White. More than 60% of adolescents reported past 30-day e-cigarette advertising exposure at each survey. Among adolescents who had never used e-cigarettes, those who reported e-cigarette advertising exposure were more likely to feel curious about using e-cigarettes (adjusted odds ratio [aOR], 1.56 [95% CI, 1.43-1.70]) and were more likely to become ever e-cigarette users (aOR, 1.21 [95% CI, 1.05-1.41]) and current e-cigarette users (aOR, 1.42 [95% CI, 1.16-1.75]) at follow-up. Adolescents who reported having best friends using e-cigarettes were more likely to feel curious about using e-cigarettes (eg, all best friends: aOR, 4.13 [95% CI, 2.35-7.26]) and initiate e-cigarette use at follow-up (eg, among adolescents reporting all best friends use e-cigarettes, risk of ever use: aOR, 4.08 [95% CI, 1.44-11.59]; risk of current use aOR, 5.42 [95% CI, 1.49-19.72]) than adolescents who reported having no best friends using e-cigarettes.

**Conclusions and Relevance:**

This cohort study of US adolescents found that e-cigarette advertising and peer influence were significantly associated with e-cigarette initiation. Efforts to address youth vaping need to consider peer influence and incorporate measures reducing e-cigarette advertising exposure.

## Key Points

**Question:**

Are exposure to e-cigarette advertising and parental and peer influence associated with e-cigarette use among US adolescents?

**Findings:**

This cohort study of a total of 11 641 adolescents from the Population Assessment of Tobacco and Health study waves 4, 4.5, and 5, found that adolescents who reported past 30-day e-cigarette advertising exposure and who reported having best friends using e-cigarettes were more likely to feel curious about using e-cigarettes and more likely to initiate e-cigarette use at follow-up.

**Meaning:**

These findings suggest that efforts to address youth vaping need to consider peer influence and incorporate measures reducing e-cigarette advertising exposure.

## Teaser

This cohort study examines how e-cigarette advertising exposure and parental and peer e-cigarette use are associated with e-cigarette use among US adolescents.

## Introduction

In 2014, e-cigarettes surpassed combustible cigarettes and became the most commonly used tobacco product among US adolescents.^1,2,3,4^ The 2021 National Youth Tobacco Survey (NYTS) estimated that more than 2 million adolescents (11.3% of high school students and 2.8% of middle school students) in the US currently used e-cigarettes.^4^ The popularity of e-cigarettes among US youth has been attributed to e-cigarette’s sleek and stealth product designs and availability of a wide varieties of flavors appealing to youth.^5,6^ Importantly, marketing has also played a critical role in popularizing e-cigarettes among adolescents.^7,8,9,10,11^ It is noteworthy that much of e-cigarette marketing has been documented by previous studies to be youth-targeted.^5,7,8^ For example, a 2017 study by Padon et al^7^ collected 154 e-cigarette video advertisements and found all advertisements included some youth-appealing content. The e-cigarette giant, JUUL, has been criticized for using young models and youth-appealing content in its social media marketing campaigns and accused of being responsible, at least in part, for the surge in youth vaping.^8,12,13,14^ Data from the NYTS showed that 78.2% of middle and high school students were exposed to e-cigarette advertising in 2016, increased from 68.9% in 2014.^15^

Previous studies have indicated that e-cigarette advertising exposure was significantly associated with e-cigarette use and susceptibility among US adolescents.^9,10,16,17,18^ Data from the 2014 NYTS showed that exposure to each channel of e-cigarette advertising (internet, print, retail, and TV and movies) was significantly associated with ever or current e-cigarette use, as well as susceptibility to use among never e-cigarette users.^16,18^ A randomized trial conducted among adolescents who were e-cigarette naive showed that exposure to e-cigarette TV advertisements increased intentions to use e-cigarettes.^17^ A 2018 study by Camenga et al^10^ used longitudinal data collected from middle and high school students in Connecticut and found that exposure to e-cigarette advertising on Facebook was associated with higher odds of subsequent e-cigarette use.^10^

e-Cigarettes have been regulated by the US Food and Drug Administration (FDA) as a tobacco product since the final deeming rule was issued in 2016.^19^ Steps have been taken to restrict youth access to e-cigarettes and curb e-cigarette marketing aimed at youth since then.^20,21,22,23,24^ It has been reported that some e-cigarette companies withdrew certain flavored products and suspended certain youth-targeted marketing activities in response to the heighted scrutiny from the FDA.^25,26^ However, changes of e-cigarette advertising exposure in the context of a rapidly changing regulatory environment have not been examined, and little is known about how e-cigarette advertising exposure was associated with e-cigarette use and susceptibility among US adolescents in recent years.

Additionally, previous studies investigating factors associated with adolescents’ e-cigarette use did not fully consider the potential influence of parental and peer e-cigarette use behaviors. Current evidence about associations of parental and peer use with tobacco use is almost entirely from studies of cigarettes and other tobacco products,^27,28^ with most of these studies focusing on the associations between adolescents’ cigarette smoking and the smoking status of their friends.^28,29,30^ A few studies have suggested that a similar pattern exists for e-cigarettes^31,32^; however, these studies relied primarily on regional data collected from convenience samples. Nationally representative studies are needed to estimate the associations between parental and peer e-cigarette use and adolescents’ e-cigarette use outcomes.

To fill these critical research gaps, this study uses data from the most recent 3 waves of the Population Assessment of Tobacco and Health (PATH) study surveys to examine trends of overall e-cigarette advertising exposure and advertising exposure from specific media channels among US adolescents and how e-cigarette advertising exposure and parental and peer e-cigarette use were associated with contemporary curiosity about using e-cigarettes and future e-cigarette initiation at follow-up among adolescents who had never used e-cigarettes. We hypothesized that e-cigarette advertising exposure was still high among US adolescents despite recent FDA regulatory actions restricting certain youth-targeted e-cigarette advertising and that e-cigarette advertising exposure and parental and peer use of e-cigarettes were significantly associated with e-cigarette use and susceptibility.

## Methods

This cohort study is a secondary analysis of the PATH study and was exempt from ethical review by the Georgia State University institutional review board because the data were deidentified. This PATH study was approved by the Westat institutional review board. Written informed consents were obtained from all study participants and parents (if applicable). This study is reported following the Strengthening the Reporting of Observational Studies in Epidemiology (STROBE) reporting guideline.

### Data

Data used in this study were from the PATH study Youth Cohort wave 4 (December 2016 to January 2018), wave 4.5 (December 2017 to December 2018), and wave 5 (December 2018 to November 2019) surveys. The PATH study is an ongoing, nationally representative longitudinal study conducted by the National Institutes of Health and the FDA. Data collection was conducted by Westat. Detailed information on the study design and sampling strategies can be found elsewhere.^33^ The weighed response rate for the wave 4 cohort was 89.1% at wave 4.5 survey, and 83.5% at wave 5 survey. Since the data collection for these 3 waves of surveys were mainly conducted in 2017, 2018, and 2019, respectively, survey year was used for each wave to show the trends of e-cigarette advertising exposure. Study sample were adolescents (age 12-17 years) with wave 4 cohort all-wave weights. Answers like “Refused” and “I don’t know” were coded as missing values, and pair-wise deletion was used to handle missing values.

### Measures

Adolescents’ e-cigarette advertising exposure was measured as past 30-day any exposure and exposure through multiple channels. Respondents were asked “In the past 30 days, have you noticed e-cigarettes or other electronic nicotine products being advertised in any of the following places? Choose all that apply.” with answer options “I haven’t seen any advertisements in the past 30 days,” “At gas stations, convenience stores, or other retail stores” (coded as *stores*), “On billboards” (coded as *billboards*), “In newspapers or magazines” (coded as *print*), “On radio” (coded as *radio*), “On television” (coded as *TV*), “At events such as fairs, festivals, or sporting events” (coded as *events*), “At nightclubs, bars, or music concerts” (coded as *clubs or bars*), “On websites or social media sites” (coded as *online*), and “Somewhere else.” No other options could be selected together with the first option. Adolescents who did not select the first option and selected any of the following options were coded as having past 30-day e-cigarette advertising exposure. Parental use was measured by parental past 30-day e-cigarette use. Peer use was measured by respondents’ reports of how many of their best friends used e-cigarettes (none, a few, some, most, or all).

The outcomes were contemporary curiosity about using e-cigarettes and e-cigarette initiation at the 12-month follow-up. Adolescents who had never used e-cigarettes were asked if they had ever been curious about using an e-cigarette. Those who answered very, somewhat, or a little were coded as yes, and those who answered not at all were coded as no. e-Cigarette initiation was measured as ever use and current (past 30-day) use at follow-up among baseline never users.

Other covariates included in this study were biological sex (boy and girl), age in years, race and ethnicity (categorized as Hispanic, non-Hispanic Black, non-Hispanic White, and other), highest parental education (less than high school, high school graduate, some college or associate degree, and bachelor’s degree and above), severity of internalizing and externalizing mental health problems (low, moderate, and high), perception of harm from e-cigarette use (no harm, little harm, some harm, and a lot of harm), current (past 30-day) cigarette smoking status,^34,35^ and current (past 30-day) use of other tobacco products (cigar, pipe, hookah, bidi, kretek, and smokeless tobacco, including snus and dissolvable products).^36,37^ Race and ethnicity, which were self-reported by study participants, were included as a covariate since previous studies have indicated that race and ethnicity are significantly associated with adolescents’ e-cigarette use behaviors.^4,5^ The other racial and ethnic groups included Asian Indian, Chinese, Filipino, Guamanian or Chamorro, Japanese, Korean, non-Hispanic American Indian or Alaska Native, Native Hawaiian, other Asian, other Pacific Islander, Samoan, and Vietnamese. Measures for adolescents’ mental health conditions were based on screening questions asking adolescents’ symptoms pertinent to mental health problems in the PATH study. Specifically, there were 4 items for internalizing symptoms and 7 items for externalizing symptoms. For each item, adolescents who had experienced significant problems with it in the past 12 months were coded as 1 and others were coded as 0. Scores for internalizing and externalizing problems were summed up respectively and categorized to low (0-1), moderate (2-3), and high (≥4) severity.^38,39^

### Statistical Analysis

Data management and analysis were conducted using Stata statistical software version 16.1 (StataCorp). Wave 4 cohort all-wave weights were applied to account for the complex sampling strategies and nonresponse rates and to generate nationally representative estimates. Prevalence of any past 30-day e-cigarette advertising exposure and exposure from the 8 channels (ie, stores, billboards, print, radio, TV, events, clubs or bars, and online) were estimated separately for the full study sample and for adolescents who had never used e-cigarettes (eFigure in the Supplement). Generalized estimating equations were used to estimate the adjusted associations between exposures and each outcome, controlling for all specified covariates, study wave, and participant’s state of residence. All statistical tests were 2-tailed with significance level set at α = .05. Data were analyzed in January 2022.

## Results

### Sociodemographic Factors of Study Sample

The sample size was 8548 participants for wave 4, 10 073 participants for wave 4.5, and 11 641 participants for wave 5. The sample size increased because youth aged 9 to 11 years aged up and joined the youth cohort at each wave (eFigure in the Supplement). Among adolescents in the wave 4 survey, 4425 (51.1%) were boys, 1935 (24.9%) were aged 12 years, 1105 (13.0%) were Black, 2515 (24.4%) were Hispanic, and 3702 (52.3%) were White. The distributions of sex and race and ethnicity did not change significantly across study waves. During the study period, the prevalence of ever e-cigarette use increased from 9.0% (95% CI, 8.4%-9.7%) of participants in wave 4 to 19.9% (95% CI, 19.1%-20.6%) of participants in wave 5. Report of parental past 30-day e-cigarette use increased from 3.9% (95% CI, 3.5%-4.4%) of participants in wave 4 to 5.2% (95% CI, 4.8%-5.6%) of participants in wave 5. Adolescents who reported none of their best friends using e-cigarettes decreased from 84.3% (95% CI, 83.4%-85.1%) of participants in wave 4 to 63.4% (95% CI, 62.5%-64.4%) of participants in wave 5 (eTable in the Supplement).

### e-Cigarette Advertising Exposure

**Figure 1.**
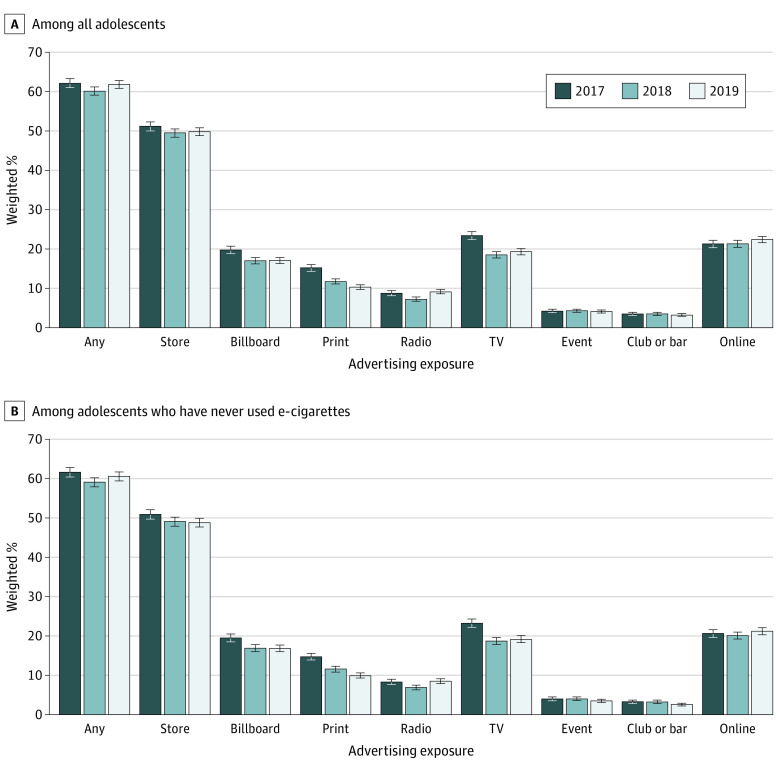
Prevalence of Past 30-Day e-Cigarette Advertising Exposure Among US Adolescents, 2017-2019

Figure 1 shows the prevalence of any e-cigarette advertising exposure and exposure from multiple channels in the past 30 days among all participating adolescents and among adolescents who had never used e-cigarettes. It was estimated that more than 60% of US adolescents reported exposure to e-cigarette advertising in the past 30 days at each survey during the study period. In 2017, exposure was highest in stores (51.2% [95% CI, 50.0%-52.3%] of participants), followed by TV (23.4% [95% CI, 22.4%-24.4%] of participants), online (21.3% [95% CI, 20.3%-22.2%] of participants), and billboards (19.7% [95% CI, 18.8%-20.7%]). Advertising exposure from stores and online did not change significantly from 2017 to 2019. Exposure from billboard and TV decreased from 2017 to 2018 but did not change significantly from 2018 to 2019. Exposure from print media decreased from 15.2% (95% CI, 14.3%-16.0%) in 2017 to 9.1% (95% CI, 8.6%-9.7%) in 2019. The prevalence of e-cigarette advertising exposure and trends were similar for adolescents who had never used e-cigarettes compared with all adolescent respondents.

### Factors Associated With Curiosity About Using e-Cigarettes

**Figure 2.**
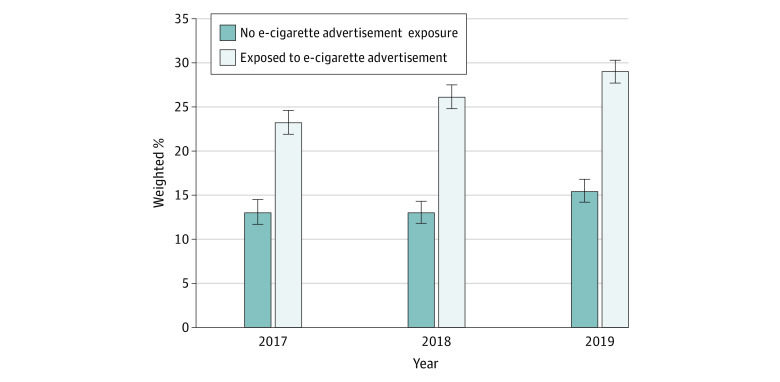
Proportion of Adolescents Feeling Curious About Using e-Cigarettes by e-Cigarette Advertising Exposure Status

Bivariate associations showed that among adolescents who had never used e-cigarettes, those who reported any past 30-day e-cigarette advertising exposure were more likely to feel curious about using e-cigarettes compared with adolescents who reported no exposure (2017: 23.2% [95% CI, 21.9%-24.6%] of participants vs 13.0% [95% CI, 11.7%-14.5%] of participants; 2018: 26.1% [95% CI, 24.8%-27.5%] of participants vs 13.0% [95% CI, 11.8%-14.3%] of participants; 2019: 29.0% [95% CI, 27.7%-30.3%] of participants vs 15.4% [95% CI, 14.2%-16.8%] of participants) (Figure 2). Adjusted regression analysis showed consistent results (adjusted odds ratio [aOR], 1.56 [95% CI, 1.43-1.70]) (Table 1).

**Table 1.** Adjusted Associations of Curiosity in Using e-Cigarettes With e-Cigarette Advertising Exposure, Parental Effect, and Peer Effect

Factor	OR (95% CI)^a^
Past 30-d any e-cigarette advertising exposure^b^	1.56 (1.43-1.70)
Parental e-cigarette use^b^	1.27 (1.03-1.56)
Friends using e-cigarettes	
None	1 [Reference]
A few	2.60 (2.36-2.86)
Some	3.40 (2.91-3.96)
Most	4.96 (3.90-6.31)
All	4.13 (2.35-7.26)
Perception of harm from e-cigarette use	
No harm	2.73 (2.00-3.72)
Little harm	3.93 (3.45-4.49)
Some harm	2.43 (2.24-2.65)
A lot of harm	1 [Reference]
Sex	
Boys	0.91 (0.83-1.00)
Girls	1 [Reference]
Age, y	
12	1 [Reference]
13	1.17 (1.03-1.33)
14	1.39 (1.23-1.58)
15	1.51 (1.33-1.73)
16	1.32 (1.13-1.55)
17	1.31 (1.07-1.61)
Race and ethnicity	
Black	0.97 (0.83-1.13)
Hispanic	1.10 (0.97-1.24)
White	1 [Reference]
Other^c^	1.03 (0.88-1.21)
Parental education	
<High school	1 [Reference]
High school graduate	0.88 (0.76-1.02)
Some college or associate degree	0.96 (0.84-1.10)
≥Bachelor’s degree	1.03 (0.90-1.19)
Severity of internalizing mental health problems	
Low	1 [Reference]
Moderate	1.31 (1.19-1.45)
High	1.57 (1.40-1.76)
Severity of externalizing mental health problems	
Low	1 [Reference]
Moderate	1.61 (1.44-1.79)
High	2.28 (2.03-2.55)
Current cigarette smoking^b^	1.43 (0.78-2.60)
Current use of other tobacco products^b^	2.05 (1.14-3.68)
Wave	
4	0.76 (0.68-0.83)
4.5	0.81 (0.75-0.88)
5	1 [Reference]

Abbreviation: OR, odds ratio.

^a^ Participant state of residence was controlled for.

^b^ Reference group was those who answered no.

^c^ Other included Asian Indian, Chinese, Filipino, Guamanian or Chamorro, Japanese, Korean, Native Hawaiian, non-Hispanic American Indian or Alaska Native, other Asian, other Pacific Islander, Samoan, and Vietnamese.

Adolescent never e-cigarette users with parents currently using e-cigarettes were more likely to feel curious about using e-cigarettes (aOR, 1.27 [95% CI, 1.03-1.56]). Compared with adolescents who reported none of their best friends using e-cigarettes, adolescents were more likely to feel curious about using e-cigarettes if they reported that a few (aOR, 2.60 [95% CI, 2.36-2.86]), some (aOR, 3.40 [95% CI, 2.91-3.96]), most (aOR, 4.96 [95% CI, 3.90-6.31]), or all (aOR, 4.13 [95% CI, 2.35-7.26]) of their best friends used e-cigarettes (Table 1).

### Factors Associated With e-Cigarette Initiation at Follow-up

**Figure 3.**
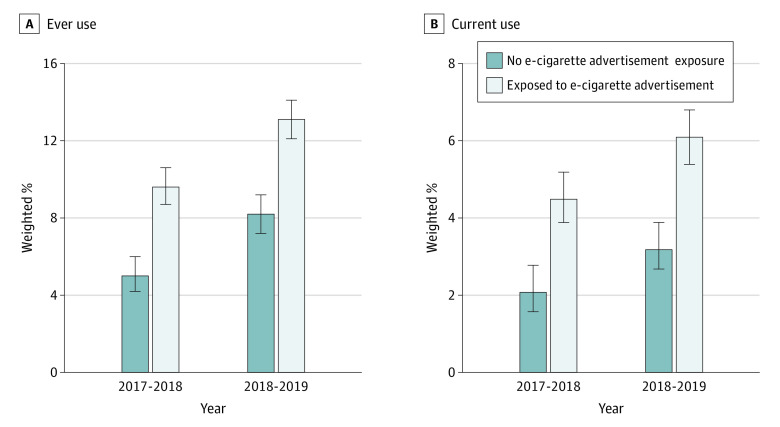
Proportion of e-Cigarette Initiation at Follow-up by Baseline e-Cigarette Advertising Exposure Status

Figure 3 shows the bivariate associations between baseline e-cigarette advertising exposure and e-cigarette initiation at follow-up among adolescents who had never used e-cigarettes at baseline. Adolescents who reported e-cigarette advertising exposure, compared with those who reported no exposure, were more likely to become ever e-cigarette users (2018: 9.6% [95% CI, 8.7%-10.6%] of participants vs 5.0% [95% CI, 4.2%-6.0%] of participants; 2019: 13.1% [95% CI, 12.1%-14.1%] of participants vs 8.2% [95% CI, 7.2%-9.2%] of participants) and current e-cigarette users (2018: 4.5% [95% CI, 3.9%-5.2%] of participants vs 2.1% [95% CI, 1.6%-2.8%] of participants; 2019: 6.1% [95% CI, 5.4%-6.8%] of participants vs 3.2% [95% CI, 2.7%-3.9%] of participants) at follow-up. Adjusted associations showed consistent results at follow-up for ever e-cigarette use (aOR, 1.21 [95% CI, 1.05-1.41]) and current e-cigarette use (aOR, 1.42 [95% CI, 1.16-1.75]) (Table 2).

**Table 2.** Adjusted Associations of e-Cigarette Initiation at Follow-up With Baseline Past 30-Day e-Cigarette Advertising Exposure, Parental Use, and Peer Use

Factor	e-Cigarette use, OR (95% CI)^a^	
	Ever	Current
Past 30-d any e-cigarette advertising exposure^b^	1.21 (1.05-1.41)	1.42 (1.16-1.75)
Parental e-cigarette use^b^	1.33 (0.96-1.85)	1.10 (0.71-1.68)
Friends using e-cigarettes		
None	1 [Reference]	1 [Reference]
A few	2.69 (2.27-3.18)	2.27 (1.81-2.85)
Some	3.69 (2.91-4.68)	3.35 (2.45-4.58)
Most	4.56 (3.14-6.63)	4.40 (2.77-7.00)
All	4.08 (1.44-11.59)	5.42 (1.49-19.72)
Perception of harm from e-cigarette use		
No harm	2.44 (1.55-3.84)	3.21 (1.78-5.80)
Little harm	2.56 (2.10-3.13)	2.01 (1.53-2.65)
Some harm	1.50 (1.30-1.73)	1.27 (1.04-1.56)
A lot of harm	1 [Reference]	1 [Reference]
Sex		
Boys	0.89 (0.78-1.02)	0.83 (0.69-0.99)
Girls	1 [Reference]	1 [Reference]
Age, y		
12	1 [Reference]	1 [Reference]
13	2.33 (1.79-3.02)	2.99 (2.00-4.45)
14	2.69 (2.09-3.47)	3.26 (2.20-4.84)
15	3.11 (2.41-4.03)	4.10 (2.77-6.08)
16	2.66 (1.98-3.59)	3.73 (2.40-5.80)
17	3.74 (1.15-12.15)	4.98 (1.20-20.61)
Race and ethnicity		
Black	0.39 (0.30-0.50)	0.33 (0.23-0.47)
Hispanic	0.70 (0.58-0.85)	0.70 (0.54-0.92)
White	1 [Reference]	1 [Reference]
Other^c^	0.62 (0.49-0.78)	0.56 (0.40-0.79)
Parental education		
Less than high school	1 [Reference]	1 [Reference]
High school graduate	0.89 (0.71-1.13)	0.91 (0.65-1.28)
Some college or associate degree	0.98 (0.79-1.20)	0.87 (0.65-1.18)
Bachelor's degree or above	0.93 (0.75-1.16)	0.79 (0.58-1.09)
Severity of internalizing mental health problems		
Low	1 [Reference]	1 [Reference]
Moderate	1.11 (0.94-1.31)	1.05 (0.83-1.31)
High	1.28 (1.06-1.54)	1.07 (0.83-1.39)
Severity of externalizing mental health problems		
Low	1 [Reference]	1 [Reference]
Moderate	1.39 (1.16-1.67)	1.29 (1.01-1.66)
High	1.83 (1.51-2.22)	1.89 (1.46-2.44)
Current cigarette smoking^b^	2.55 (1.29-5.06)	2.26 (0.91-5.59)
Current use of other tobacco products^b^	3.42 (1.67-6.99)	4.80 (2.09-11.05)
Wave period		
4 to 4.5	0.78 (0.68-0.89)	0.83 (0.69-1.01)
4.5 to 5	1 [Reference]	1 [Reference]

Abbreviation: OR, odds ratio.

^a^ Participant state of residence was also controlled for.

^b^ Reference group was those who answered no.

^c^ Other included Asian Indian, Chinese, Filipino, Guamanian or Chamorro, Japanese, Korean, Native Hawaiian, non-Hispanic American Indian or Alaska Native, other Asian, other Pacific Islander, Samoan, and Vietnamese.

Compared with those who reported none of their best friends using e-cigarettes, baseline never e-cigarette users were more likely to become ever users if they reported a few (aOR, 2.69 [95% CI, 2.27-3.18]), some (aOR, 3.69 [95% CI, 2.91-4.68]), most (aOR, 4.56 [95% CI, 3.14-6.63]), or all (aOR, 4.08 [95% CI, 1.44-11.59]) of their best friends used e-cigarettes. Similar findings were found for risk of becoming current users among adolescents who reported a few (aOR, 2.27 [95% CI, 1.81-2.85]), some (aOR, 3.35 [95% CI, 2.45-4.58]), most (aOR, 4.40 [95% CI, 2.77-7.00]) or all (aOR, 5.42 [95% CI, 1.49-19.72]) of their best friends used e-cigarettes (Table 2).

## Discussion

This cohort study using nationally representative longitudinal data provides important evidence on trends of e-cigarette advertising exposure from 2017 to 2019 and on how e-cigarette advertising and parental and peer e-cigarette use were associated with e-cigarette initiation and susceptibility among US adolescents. Our study results show that, despite measures taken to regulate e-cigarette companies’ youth-targeted marketing, e-cigarette advertising exposure was still high among US adolescents, regardless of their e-cigarette use status, and e-cigarette advertising exposure and peer use of e-cigarettes were significantly associated with contemporary curiosity about using e-cigarettes and future e-cigarette initiation among adolescents who had never used e-cigarettes.

Consistent with previous findings,^10,15^ we also found that e-cigarette advertising exposure was highest at point-of-sale (ie, stores: 49.8% of participants in 2019), suggesting the presence of a high level of e-cigarette marketing by e-cigarette companies in retail settings. In addition, our study found that TV, online, and billboards were also major sources of e-cigarette advertising exposure among US youth. Importantly, although overall e-cigarette advertising exposure, and exposure from TV, billboards, and print media decreased from 2017 to 2018, the overall level of e-cigarette advertising exposure in 2019 was comparable with that in 2017. In addition, despite the FDA’s intense regulatory scrutiny of e-cigarette companies’ advertising on social media, online e-cigarette advertising exposure did not change significantly during our study period. The decrease in e-cigarette advertising from 2017 to 2018 was likely due to the heightened federal regulations.^21,23,24^ For example, it was reported that, beginning in early 2018, the US FDA and the Federal Trade Commission issued multiple warning letters to e-cigarette companies and retailers for promoting e-cigarette products in ways misleading to youth or selling e-cigarette products to youth illegally.^23,40^ The FDA also took actions to prevent youth access to e-cigarette products.^21,23,24,40,41^ However, despite these efforts, our study findings suggest that the level of exposure to e-cigarette advertising, both overall and via specific channels (eg, online), was still high among adolescents in our study period. More targeted efforts may be needed to reduce youth e-cigarette advertising exposure.

Our study also found that adolescent never e-cigarette users who reported any past 30-day e-cigarette advertising exposure were significantly more likely to feel curious about using e-cigarettes and more likely to initiate e-cigarette use at 1-year follow-up. Existing literature has documented the powerful influence associated with e-cigarette advertising in increasing adolescents’ intentions to use and use of e-cigarettes.^9,10,16,17^ Our findings demonstrated that even in a context of intensified regulatory environment with heightened scrutiny of youth-targeted e-cigarette marketing, e-cigarette companies could still find ways to get around these regulations to market their products to adolescents, which in turn may increase adolescents’ e-cigarette use and susceptibility. These findings suggest that stronger and more comprehensive regulations may be needed to reduce e-cigarette advertising exposure and prevent e-cigarette initiation for US adolescents. The World Health Organization Framework Convention on Tobacco Control called for a comprehensive ban on tobacco advertising, promotion, and sponsorship in 2003.^42^ In 2014, the World Health Organization specifically called for restrictions on e-cigarette marketing to protect the health of youth and nonsmokers.^43^ Policies that regulate youth-oriented e-cigarette marketing would benefit from incorporating the comprehensive approaches proposed by the Framework Convention on Tobacco Control.

Peer influence is one of the key factors that can affect adolescent cigarette smoking.^28,29,30^ Our study also found that having best friends using e-cigarettes was associated with higher odds of e-cigarette initiation and susceptibility, consistent with what has been reported previously using convenience samples.^31,32^ According to multiple health theories and models,^44,45,46^ perceived peer approval and use of substances play key roles in influencing early stages of substance use during adolescence. Our findings indicate that these theories and models are also applicable to e-cigarette use. These findings suggest that efforts to change social norms toward e-cigarettes and to communicate the risks of e-cigarette use during adolescence are needed as part of a comprehensive strategy to reverse the evolving trend of youth vaping in the US.

Contrary to our initial hypothesis, we found that baseline parental e-cigarette use was not significantly associated with adolescents’ e-cigarette initiation at 1-year follow-up, suggesting youth vaping behaviors may be more in sync with the behavior of their peers than their parents.^6,47,48^ This finding may be explained in part by the source of e-cigarettes for youth, which came primarily from their peers.^48^ Additionally, the small sample percentage of parents who used e-cigarettes may be also a factor.

### Limitations

This study has some limitations. First, this study used self-reported data, which may induce recall bias and social desirability bias. Second, despite the temporal association, this study design could not establish a causal relationship. Third, although we controlled for a variety of covariates, other potential confounders may still exist and were not accounted for in our models. Furthermore, this study conducted multiple hypothesis tests without α correction.

## Conclusions

This cohort study found that e-cigarette advertising exposure was high among US adolescents between 2017 and 2019, regardless of their e-cigarette using status. e-Cigarette advertising exposure and peer use of e-cigarettes were significantly associated with contemporary curiosity about using e-cigarettes and future e-cigarette initiation among adolescents who had never used e-cigarettes. Efforts to address the increasing use of vaping among youth need to consider peer influence and incorporate measures reducing e-cigarette advertising exposure.

## Notes

Supplement. **eFigure.** Flowchart of Analytical Sample **eTable.** Descriptive Statistics of Study Sample
