# Supplementary material for: Association of e-Cigarette Advertising, Parental Influence, and Peer Influence With US Adolescent e-Cigarette Use
Source: JAMA Netw Open. 2022 Sep 29;5(9):e2233938. doi: 10.1001/jamanetworkopen.2022.33938 (PMC9523494; doi:10.1001/jamanetworkopen.2022.33938)
Supplement: Supplement. — eFigure. Flowchart of Analytical Sample eTable. Descriptive Statistics of Study Sample [file jamanetwopen-e2233938-s001.pdf]

Supplemental Online Content

Wang Y, Duan Z, Weaver SR, et al. Association of e-cigarette advertising, parental influence, and peer influence with US adolescent e-cigarette use. *JAMA Netw Open*. 2022;5(9):e2233938. doi:10.1001/jamanetworkopen.2022.33938

eFigure. Flowchart of Analytical Sample

eTable. Descriptive Statistics of Study Sample

This supplemental material has been provided by the authors to give readers additional information about their work.

© 2022 Wang Y et al. *JAMA Network Open.*

eFigure 1. Flowchart of Analytical Sample

eFigure 1a. Analytical sample that involves all adolescent participants across the PATH study Wave 4, 4.5 and 5

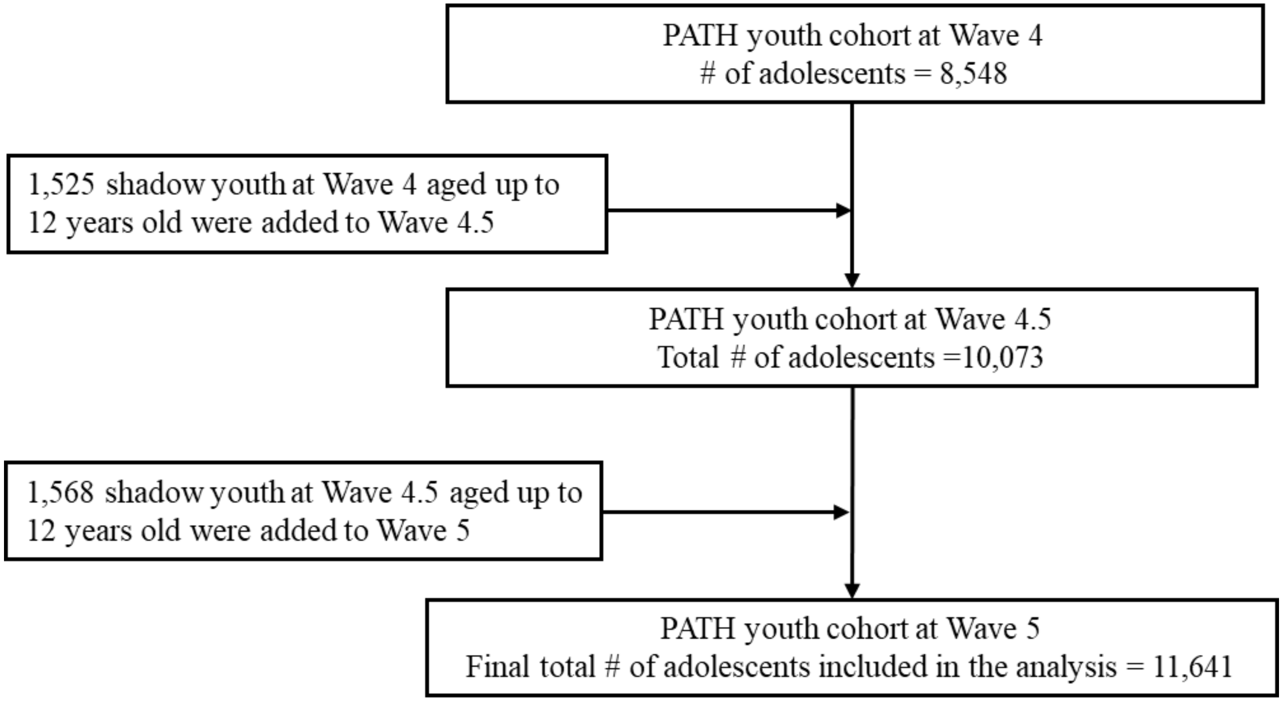

© 2022 Wang Y et al. *JAMA Network Open.*

eFigure 1b. Analytical sample that involves adolescent never e-cigarette users across the PATH study Wave 4, 4.5, and 5

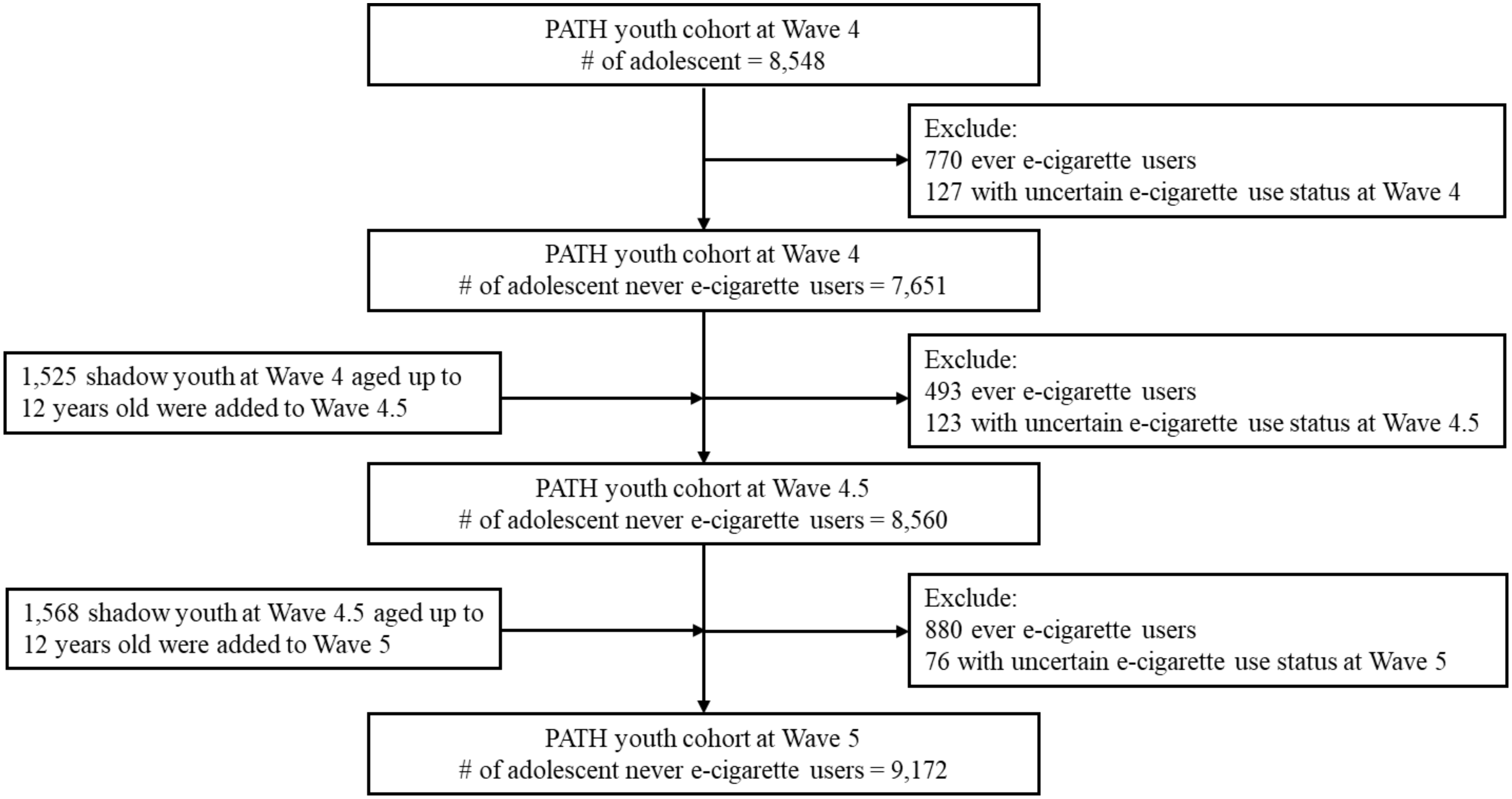

Note: Adolescent never e-cigarette users were used to estimate the prevalence of e-cigarette advertising exposure among this subgroup, and the contemporary association between exposures and curiosity in using e-cigarettes. Adolescent e-cigarette never users at baseline waves (Wave 4 for Wave 4-4.5 analysis, and Wave 4.5 for Wave 4.5-5 analysis) were used to examine the longitudinal associations between exposures and e-cigarette initiation at 1-year follow-up.

© 2022 Wang Y et al. *JAMA Network Open.*

eTable 1. Descriptive Statistics of Study Sample

	Wave 4 (N = 8,548)		Wave 4.5 (N = 10,073)		Wave 5 (N = 11,641)	
	**n (weighted %)**	**95% CI**	**n (weighted %)**	**95% CI**	**n (weighted %)**	**95% CI**
Sex						
Boy	4,425 (51.1%)	50.0 - 52.2	5,225 (51.1%)	50.0 - 52.2	6,063 (51.1%)	50.1 - 52.1
Girl	4,090 (48.9%)	47.8 - 50.0	4,810 (48.9%)	47.8 - 50.0	5,531 (48.9%)	47.9 - 49.9
Age in years						
12	1,935 (24.9%)	23.9 - 25.9	1,523 (19.5%)	18.6 - 20.5	1,624 (17.3%)	16.5 - 18.1
13	2,179 (24.7%)	23.7 - 25.7	1,985 (20.5%)	19.7 - 21.4	1,474 (15.7%)	14.9 - 16.5
14	2,232 (24.8%)	23.8 - 25.8	2,196 (20.0%)	19.2 - 20.9	2,030 (17.5%)	16.7 - 18.2
15	2,074 (24.1%)	23.2 - 25.1	2,226 (19.8%)	19.0 - 20.7	2,181 (16.4%)	15.8 - 17.2
16	128 (1.5%)	1.3 - 1.9	2,067 (19.3%)	18.5 - 20.1	2,217 (16.6%)	15.9 - 17.3
17	-	-	76 (0.8%)	0.6 – 1.0	2,115 (16.5%)	15.8 - 17.2
Race/ethnicity						
Black	1,105 (13.0%)	12.2 - 13.8	1,245 (12.7%)	12.0 - 13.4	1,405 (12.7%)	12.1 - 13.5
Hispanic	2,515 (24.4%)	23.4 - 25.3	2,917 (24.7%)	23.8 - 25.5	3,375 (25.0%)	24.2 - 25.9
White	3,702 (52.3%)	51.2 - 53.5	4,453 (51.9%)	50.8 - 53.0	5,185 (51.4%)	50.4 - 52.5
Other[a]	817 (10.3%)	9.6 - 11.1	970 (10.7%)	10.0 - 11.4	1,111 (10.8%)	10.1 - 11.5
Parental education						
Less than high school	1,582 (15.9%)	15.1 - 16.7	1,724 (14.8%)	14.1 - 15.5	2,000 (15.2%)	14.6 - 15.9
High school graduate	1,506 (16.7%)	15.9 - 17.5	1,778 (17.0%)	16.3 - 17.9	2,040 (16.8%)	16.0 - 17.5
Some college or associate degree	2,665 (30.9%)	29.8 - 32.0	3,148 (31.0%)	30.0 - 32.0	3,617 (30.9%)	30.0 - 31.9
Bachelor's degree or above	2,727 (36.6%)	35.4 - 37.7	3,318 (37.2%)	36.1 - 38.2	3,907 (37.1%)	36.1 - 38.0
Severity of internalizing mental health problems						
Low	4,247 (49.9%)	48.7 - 51.0	4,885 (49.2%)	48.1 - 50.2	5,381 (47.3%)	46.3 - 48.3
Moderate	2,370 (28.6%)	27.6 - 29.7	2,746 (28.0%)	27.1 - 29.0	3,103 (28.0%)	27.1 - 28.9
High	1,834 (21.5%)	20.6 - 22.5	2,318 (22.8%)	21.9 - 23.7	2,840 (24.7%)	23.9 - 25.6
Severity of externalizing mental health problems						
Low	3,350 (38.9%)	37.8 - 40.0	4,108 (41.2%)	40.1 - 42.2	4,674 (41.2%)	40.3 - 42.2
Moderate	2,389 (29.3%)	28.3 - 30.4	2,707 (27.9%)	27.0 - 28.9	3,128 (27.8%)	26.9 - 28.7
High	2,648 (31.8%)	30.7 - 32.8	3,077 (30.9%)	29.9 - 31.9	3,454 (30.9%)	30.0 - 31.9
Parental e-cigarette use						
Yes	330 (3.9%)	3.5 - 4.4	353 (3.7%)	3.3 - 4.1	599 (5.2%)	4.8 - 5.6
No	8,159 (96.1%)	95.6 - 96.5	9,618 (96.3%)	95.9 - 96.7	10,970 (94.8%)	94.4 - 95.2
Friends using e-cigarettes						
None	7,165 (84.3%)	83.4 - 85.1	7,329 (73.3%)	72.4 - 74.2	7,270 (63.4%)	62.5 - 64.4

© 2022 Wang Y et al. *JAMA Network Open.*

	Wave 4 (N = 8,548)		Wave 4.5 (N = 10,073)		Wave 5 (N = 11,641)	
	**n (weighted %)**	**95% CI**	**n (weighted %)**	**95% CI**	**n (weighted %)**	**95% CI**
A few	939 (11.0%)	10.3 - 11.7	1,652 (16.5%)	15.7 - 17.3	2,549 (21.8%)	21.0 - 22.6
Some	254 (3.1%)	2.7 - 3.6	609 (6.0%)	5.5 - 6.5	977 (8.2%)	7.7 - 8.8
Most	107 (1.3%)	1.1 - 1.6	332 (3.4%)	3.0 - 3.8	642 (5.4%)	5.0 - 5.9
All	30 (0.3%)	0.2 - 0.4	79 (0.8%)	0.7 - 1.1	142 (1.1%)	0.9 - 1.3
Perception of harm from e-cigarette use						
No harm	157 (1.8%)	1.5 - 2.1	213 (2.1%)	1.8 - 2.4	169 (1.4%)	1.2 - 1.6
Little harm	850 (10.2%)	9.5 - 10.9	1,020 (10.3%)	9.7 – 11.0	1,088 (9.3%)	8.7 - 9.9
Some harm	2,558 (30.3%)	29.3 - 31.4	3,112 (31.1%)	30.1 - 32.1	3,413 (29.0%)	28.1 - 29.9
A lot of harm	4,912 (57.6%)	56.5 - 58.8	5,626 (56.5%)	55.5 - 57.6	6,909 (60.3%)	59.3 - 61.3
Ever e-cigarette use						
Yes	770 (9.0%)	8.4 - 9.7	1,390 (13.5%)	12.8 - 14.2	2,393 (19.9%)	19.1 - 20.6
No	7,651 (91.0%)	90.3 - 91.6	8,560 (86.5%)	85.8 - 87.2	9,172 (80.1%)	79.4 - 80.9
Current e-cigarette use						
Yes	193 (2.4%)	2.0 - 2.7	528 (5.2%)	4.8 - 5.7	1049 (8.7%)	8.1 - 9.2
No	8,324 (97.6%)	97.3 - 98.0	9,520 (94.8%)	94.3 - 95.2	10,549 (91.3%)	90.8 - 91.9
Current cigarette smoking						
Yes	115 (1.3%)	1.1 - 1.6	212 (2.0%)	1.7 - 2.3	288 (2.3%)	2.0 - 2.6
No	8,421 (98.7%)	98.4 - 98.9	9,844 (98.0%)	97.7 - 98.3	11,339 (97.7%)	97.4 - 98.0
Current use of other tobacco products						
Yes	94 (1.2%)	1.0 - 1.5	153 (1.5%)	1.3 - 1.8	199 (1.6%)	1.4 - 1.9
No	8,343 (98.8%)	98.5 - 99.0	9,787 (98.5%)	98.2 - 98.7	11,298 (98.4%)	98.1 - 98.6

[a] Other included non-Hispanic American Indian or Alaska Native, Asian Indian, Chinese, Filipino, Japanese, Korean, Vietnamese, other Asian, Native Hawaiian, Guamanian or Chamorro, Samoan, and other Pacific Islanders.

© 2022 Wang Y et al. *JAMA Network Open.*
